# Impact of Habitat Transformation on Soil Microbial Diversity and Functionality in Karst Mountainous Parks: A Comparative Study of Remnant Forests and Artificial Green Spaces

**DOI:** 10.1002/ece3.72021

**Published:** 2025-08-14

**Authors:** Chunhua Cen, Weize Wang, Mengping Jian, Zijin Wang, Jingyi Yang

**Affiliations:** ^1^ College of Forestry Guizhou University Guiyang China; ^2^ Guizhou Guiyang Urban Ecosystem Observation and Research Station National Forestry and Grassland Administration Guiyang China

**Keywords:** diversity, functional genes, habitat transformation, Karst mountainous park, microbial networks, soil microbe

## Abstract

The structure of microbial communities is deeply influenced by the nature of urban green spaces. Our research evaluated the effects of habitat transformation on soil microbial species composition, functional gene diversity, and microbial co‐occurrence networks across three urban parks in Guiyang, spanning natural to semi‐natural environments. Bacterial evenness, as indicated by Pielou's index, was elevated in artificial green spaces, whereas bacterial and archaeal richness were substantially higher in remnant forests. This disparity underscores a crucial shift in microbial diversity linked with urbanization and landscape management. Common bacterial, fungal, and archaeal species were present across all sites, yet specific taxa distribution varied significantly with habitat type. Remnant forests harbored a greater abundance of functional genes associated with virulence factors and potential pathogenic impacts. In contrast, artificial green spaces contained a higher prevalence of genes involved in metabolic pathways, carbohydrate‐activation, and antibiotic resistance, suggesting a shift toward functional adaptations that possibly compensate for the reduced microbial species richness in more managed environments. Moreover, the complexity of microbial co‐occurrence networks was notably greater in remnant forests than in artificial green spaces, reflecting an enhanced interconnectivity that supports robust ecosystem resilience and functionality. These findings emphasize that while artificial green spaces can foster communities with greater metabolic flexibility, they do so at the cost of reduced overall microbial richness and abundance. This reduction potentially undermines ecosystem microbial diversity and ecological connectivity. Therefore, our recommendations for park management include focusing on preserving native vegetation in remnant forests and reducing organic soil amendments or chemical inputs.

## Graphical abstract

Urbanization significantly reshapes the structure of microbial communities within urban parklands, as shown in our study comparing natural to semi‐natural environments across Guiyang's green spaces. Our findings indicate a stark contrast in microbial richness and functional diversity between remnant forests and artificial green spaces, with forests showing greater species richness and network complexity, which suggests better ecosystem resilience. To enhance ecosystem health in urban parks, management practices should prioritize the conservation of remnant forests and improve microbial diversity in artificially maintained areas.

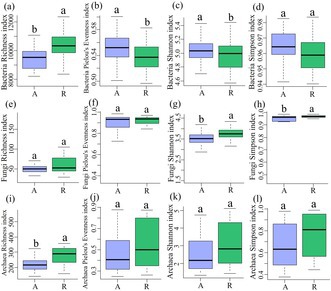

## 1 Introduction

Urban environments, which now dominate terrestrial landscapes, increasingly rely on green spaces to sustain human health amidst burgeoning urban populations (Nieuwenhuijsen et al. 2017). These spaces are critical for biodiversity, which has been shown to support human health (Twohig‐Bennett and Jones 2018; Stanhope et al. 2020). Urban green spaces are essential for promoting physical activity, mitigating environmental hazards, alleviating mental stress, and enhancing microbial diversity in living environments (Yang, Zhao, et al. 2021). Microbial diversity not only sustains various ecosystem functions but also regulates the immune system, representing an ecosystem service that is frequently overlooked and undervalued (Rook 2013). However, suboptimal land management practices and environmental changes are degrading below‐ground communities globally, leading to a decrease in soil biodiversity that impairs overall ecosystem health (Wall et al. 2015).

Microbial diversity is crucial for biodiversity, particularly within soil‐based bacteria and fungi which underpin biogeochemical cycles (Bar‐On et al. 2018). These soil microorganisms are pivotal for ecosystem health and functionality, playing key roles in material cycling, organic matter decomposition, regulating plant growth, and enhancing soil fertility (Chaparro et al. 2012). Urban green spaces, through the preservation of diverse microbial and faunal communities, can mitigate the adverse effects of urbanization and help maintain microbial species diversity (Li et al. 2018). However, the transformation of natural forests into artificial green areas in urban settings modifies the original species composition and community structure (Li et al. 2024). Introducing or overplanting non‐native plant species can disrupt nutrient cycling, negatively affecting native plant growth and overall soil ecological equilibrium (Rupprecht et al. 2015). Changes in plant composition influence soil microbial habitats, which in turn affect microbial species composition and diversity (Huo et al. 2023). Anthropogenic activities, such as urban residential developments and park management, can change soil properties like porosity and aeration, thus impacting the diversity and vigor of soil microbial communities (Kim et al. 2013; Cho et al. 2017).

Guiyang, the capital of Guizhou Province in China, is influenced by karst topography and geomorphology. Consequently, numerous mountainous remnant forests have been preserved and now surround the city, playing a crucial role in its ecological protection (Yang, Luo, et al. 2021; Liao et al. 2022). However, Guiyang's rapid urbanization poses significant threats to these forest resources, primarily through habitat loss and fragmentation (Huang et al. 2013). In response, many of these forest remnants have been integrated into the urban green space network (Luo et al. 2022). These forest parks contain natural and semi‐natural elements of remnant forests and artificial green spaces, help mitigate the erosion of forest areas by urbanization, and support biodiversity conservation in urban ecosystems (Kowarik and Lippe 2018). Transforming these remnant urban forests into parks not only expands recreational and leisure opportunities for residents but also provides a verdant refuge that contributes to stress alleviation and mental well‐being (Thompson et al. 2016). Moreover, introducing new plant species into these parks promotes greater biodiversity (Ren et al. 2017). However, the transition from remnant forests to artificial green spaces modifies the structure of the original plant communities (Wang, Gao, et al. 2024), which in turn affects soil properties including soil moisture, pH levels, and nutrient content, further impacting the native biological communities (Jeremiah et al. 2023). Native communities respond to changes in soil moisture, pH, and nutrients (Zahn et al. 2016; Oliverio et al. 2020). Although urban parks often feature more diverse soil communities, they may also exhibit greater homogeneity compared to neighboring forest soils (DelgadoBaquerizo et al. 2021). These natural and semi‐natural combinations provide an excellent opportunity to study the effects of karst mountain park transformation on soil microbes.

The types of urban green spaces alter microbial community aggregation and symbiotic networks (Wang, Feng, et al. 2024). Urban green space types significantly influence the composition of interstratified microbial communities, promote antibiotic resistance, and affect microbial aggregation (Huang et al. 2023). Antibiotic resistance genes in the soil environment represent a significant threat to human health, as they can be transferred from soil to plants, animals, and humans (Zhu et al. 2019). Moreover, changes in land use, such as the conversion of natural landscapes to urban areas, can markedly impact soil quality and the corresponding soil microbial communities (Fan et al. 2022). Alterations in soil microbial‐mediated carbon and nitrogen cycling following the transformation of remnant forests to artificial green space, where microbial communities with greater metabolic flexibility may be present, and this enhanced microbial activity may lead to increased nitrogen losses (Yang et al. 2024). Despite these insights, there remains a significant knowledge gap concerning how the transformation of remnant forests into artificial green spaces specifically alters microbial species and functional diversity. The juxtaposition of remnant forest and artificial green space within parks provides a distinctive environment for studying how anthropogenic transformation of green spaces affects soil microbial species composition and functional genetic alterations.

In this study, three mountain parks in Guiyang were selected, each categorized into two distinct habitats: remnant forests and artificial green spaces. This study aims to investigate the changes in soil microbial species and functional genes after the transformation of remnant forest into artificial green space in karst mountainous cities. By analyzing soil microbial diversity and functional genes in two habitats, it reveals the impact of mountain park transformation on microorganisms and provides key scientific evidence for the sustainable planning of karst mountain parks. (1) Differences in microbial species composition and diversity between the three parks and two habitat types were assessed; (2) differences in microbial functional genes between the two habitat types were explored; and (3) changes in microbial interactions after habitat transformation in karst mountain parks were understood by analyzing microbial co‐occurrence networks. We hypothesized that converting remnant forests into artificial green spaces would lead to reductions in soil microbial species diversity and network complexity of microbial co‐occurrence. Additionally, we anticipated a decrease in the diversity of virulence factors and pathogen‐host interaction functional genes, coupled with an increase in the abundance of genes related to metabolism and resistance. This transformation is expected to favor microbial communities that possess a broad range of metabolic capabilities, enabling them to thrive under altered environmental conditions.

## 2 Materials and Methods

### 2.1 Study Area

The study was conducted in Guiyang, the capital of Guizhou Province in southwestern China, which is situated at an average altitude of approximately 1100 m. The city experiences a subtropical humid temperate climate, with an annual average temperature of 15.3°C and precipitation of 1046 mm. The primary vegetation is subtropical evergreen deciduous broad‐leaved mixed forests. The region's plateau mountain terrain has allowed for the preservation of substantial mountain forests amidst urban development. The increasing urban demand for outdoor recreational activities has led to the conversion of many of these forests into artificial green spaces. These developments often incorporate existing mountainous features to create public parks that blend remnant forests with fabricated green areas. The study examined three such parks, each renovated at different times: Qianlingshan Park (1957), Huaguoyuan Park (2010), and Yuelianghu Park (2020), which were used as research objects (Table S1).

### 2.2 Sampling Design and Soil Collection

**FIGURE 1:**
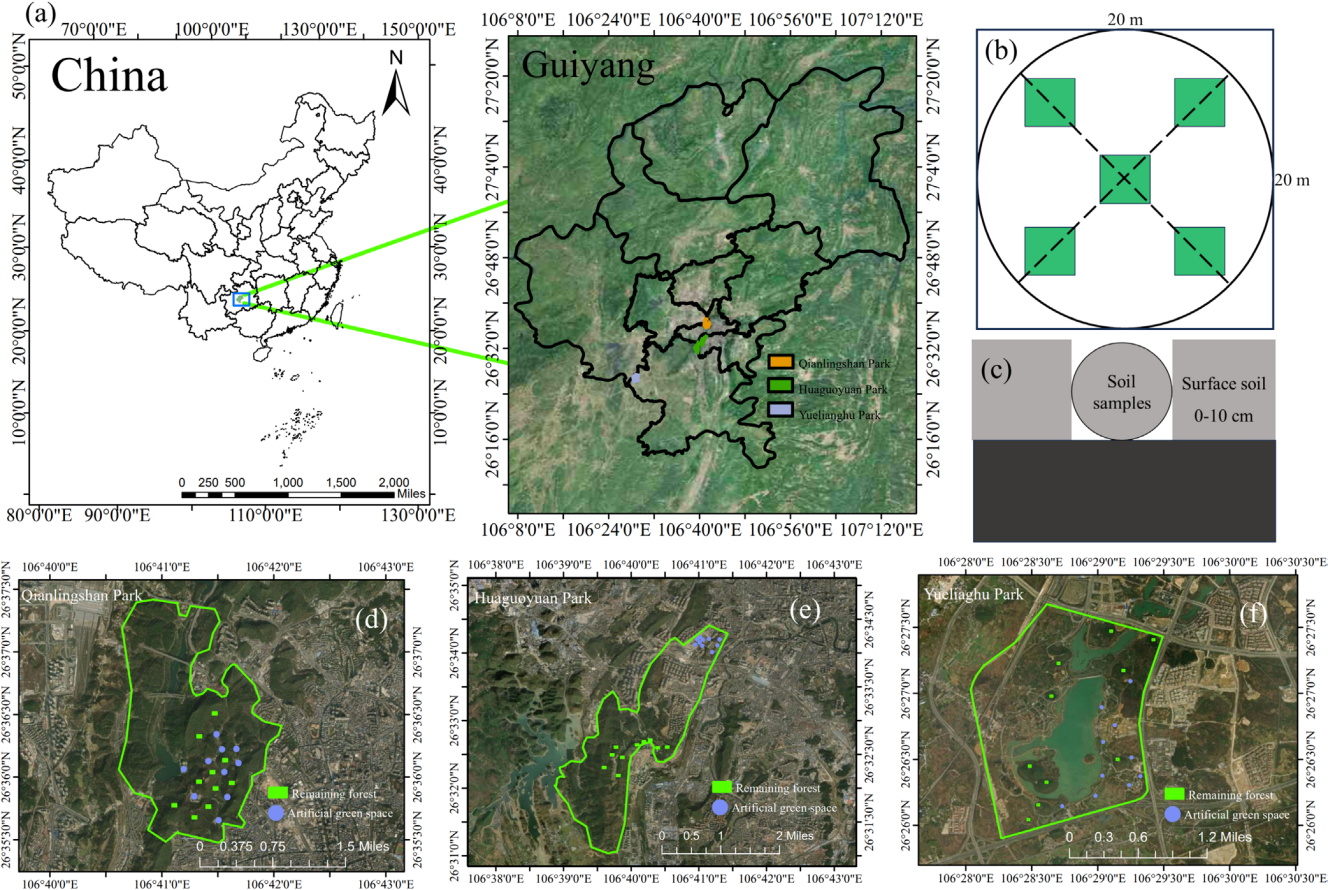
(a) Overview of the study area; (b, c) specific sampling range and method; (d) distribution of sampling points in Qianlingshan Park; (e) distribution of sampling points in Huaguoyuan Park; and (f) distribution of sampling points in Yuelianghu Park.

The three parks, originating from transformed mountain remnant forests, encompass both artificial green spaces and remnant forest habitats. This diversity facilitated the formation of six distinct groups for sampling purposes. Within these categories, ten randomly established sample plots, each measuring 20 × 20 m, were established in both the artificial and remnant sections of every park, totaling 60 plots across all parks (Figure 1d–f). In July 2023, a sampling methodology was employed whereby five‐point soil samples were collected from the top 10 cm of each plot (Figure 1b,c). These samples were combined to form a single composite sample, resulting in 60 composite samples collectively. These were then promptly transported to the laboratory for analysis within 12 h of collection.

### 2.3 DNA Extraction, Library Construction, and Metagenomics Sequencing

We extracted microbial DNA from 0.25 g fresh soil samples using the Tiangen DP 705 Kit for soil. After extraction, soil DNA was sequenced using the TGuide S96 Automated Nucleic Acid Extraction and Purification Instrument, following its specific procedure. The concentration of the resulting nucleic acids was measured using a Qubit TM dsDNA HS Assay Kit using a Qubit 3.0 fluorometer (manufactured by Invitrogen). DNA quality was also verified by assessing its integrity by 1% agarose gel electrophoresis, establishing appropriate thresholds: the concentration must be at least 5 ng/μL, the total yield must not be less than 0.5 μg, and the major integrity bands must be clearly visible above 5 kb, with no obvious impurity bands below, indicating optimal nucleic acid. Library construction for sequencing was performed using the VAHTS Universal Plus DNA Library Prep Kit for Illumina ND 617, adhering strictly to the guidelines of the manual. After construction, library fragments were checked for size using the Qsep‐400 Bioanalyser and Qubit 3.0 was again used to quantify library concentration. Qualifying parameters for the libraries included a concentration of at least 1 ng/μL, fragment size centroids between 430 and 530 bp with an average range of 420–580 bp, and peak shapes along a normal distribution and without non‐specific peaks. Sequencing of the validated libraries was performed using the Illumina NovaSeq 6000 platform utilizing the paired‐end 150 (PE 150) sequencing method.

### 2.4 Metagenomic Quality Control and Assembly

Trimmomatic software (version 0.33) was used to filter raw tags in order to provide high‐quality sequencing data. The following parameters were used: PE DIRECTOR: 3 ATTACHING: 3 SLIDINGWINDOW: 20:50 MIN120 LEN. MEGAHIT software (version 1.1.2), which omits sequences shorter than 300 base pairs, was used to assemble the macro‐genome. Next, the assembly quality was evaluated with QUAST (version 2.3) software.

### 2.5 Taxonomy and Functional Annotation

Using MetaGeneMark software (version 3.26) (http://exon.gatech.edu/meta_gmhmmp.cgi, Version 3.26) (Noguchi et al. 2006), coding areas in the genomic sequence were found. By using MMseq2 (https://github.com/soedinglab/mmseqs2, Version 11‐e1a1c) with a similarity criterion of 95% and a coverage level of 90%, sequence redundancy was eliminated (Li et al. 2015). Read counts of each gene per sample were then mapped to the gene catalog using SOAPaligner v2.2.1 (http://soap.genomics.org.cn/soapaligner.html). Based on the taxonomic annotation obtained from the NR database, 96.7% ± 0.9% of annotated sequences were assigned to bacteria, 1.9% ± 0.9% to fungi, and 0.9% ± 0.2% to archaea. After that, the protein sequences of the non‐redundant genes were compared with the NCBI NR database by BLAST using Diamond software (http://www.diamondsearch.org/index.php, version 0.8.35) (with an expected *e*‐value of 1e‐5), and the most similar sequences in the NR database were found. The corresponding annotation information is the annotation information of the corresponding sequenced genome gene. A similar method (http://www.genome.jp/kegg/) was used for functional annotation of the following databases: Kyoto Encyclopedia of Genes and Genomes (KEGG), Biologically Immediate Homologous Gene Clusters database (eggNOG), CAzy (Carbohydrate‐active enzymes database), Comprehensive Antibiotic Research Database (CARD), Virulence factor database (VFDB), and Pathogen Host Interactions Database (PHI).

### 2.6 Statistical Analysis

We used the package *vegan* to calculate alpha diversity, beta diversity, community composition of microbial species, and beta diversity analysis based on the Bray–Curtis distance as the principal coordinate analysis (PCoA). Microbial α‐diversity was quantified using the species richness index, Simpson's index, Shannon–Wiener's index, and Pielou's evenness index (Dixon 2003). We assessed differences in alpha diversity of microbial species between the two habitat types using the Wilcoxon signed‐rank test. Additionally, we evaluated variations in the top 10 functional genes in terms of relative microbial abundance between the two habitats using paired Student's *t*‐tests. Results were deemed statistically significant at *p* < 0.05. We used linear discriminant analysis of effect size (LEfSe) to identify taxa that differed significantly across habitats, regardless of taxonomic rank (Segata et al. 2011). In addition, we calculated several network topological features of soil microorganisms in artificial green spaces and remnant forests using the R packages igraph and psych. Network visualizations were created by Gephi (http://gephi.github.io/). Visualizations were performed using the ggplot2 package. Statistical analyses and visualizations were performed using R version 4.4.0 (R Core Team 2021).

## 3 Result

### 3.1 Microbial Taxonomic α‐ and β‐Diversity

**FIGURE 2:**
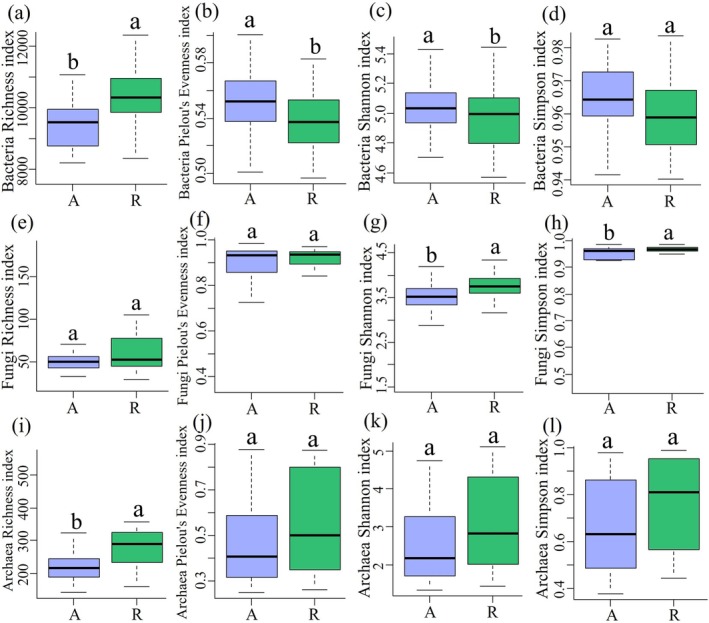
Analysis of the α‐diversity of bacteria, fungi and archaea in remnant forests and artificial green spaces. (a–d) Bacteria; (e–h) fungi; (i–l) archaea. R represents remnant forests; A represents artificial green spaces.

Bacterial and archaeal richness was significantly higher in the remnant forests than in the artificial green spaces, whereas fungal richness was not significantly different (Figure 2a,e,i). In contrast, the bacterial Pielou's evenness index was significantly higher in the artificial green spaces than in the remnant forests, whereas the difference between the fungal and archaeal Pielou's evenness index was not significant (Figure 2b,f,j). Similarly, the bacterial Shannon index was elevated in the artificial green spaces compared to the remnant forests, whereas the fungal Shannon index was significantly higher in the remnant forests than in the artificial green spaces (Figure 2c,g,k). Furthermore, the Simpson index for fungi was significantly higher in the remnant forests than in the artificial green spaces (Figure 2d,h,l).

Analyzing bacterial and archaeal diversity in six assemblages from two habitats across three parks revealed notable variations. Specifically, the remnant forests in Huaguoyuan Park exhibited a significantly higher bacterial and archaeal richness compared to its artificial green spaces. Archaeal richness was notably higher in the remnant forests of Qianlingshan Park. The bacterial Pielou's evenness index was significantly greater in the artificial green spaces of Yuelianghu Park than in the remnant forests of Huaguoyuan Park. Moreover, the archaeal Pielou's evenness index in Yuelianghu Park significantly surpassed those in Qianlingshan and Huaguoyuan Parks. The Shannon index for the remnant forests in Yuelianghu Park significantly exceeded that in the artificial green spaces of Huaguoyuan Park. Conversely, the bacterial Simpson index was higher in the artificial green spaces of Yuelianghu Park compared to the remnant forests of Huaguoyuan Park, and the archaeal Simpson index was significantly higher in the remnant forests of Qianlingshan Park than in its artificial green area. Despite these differences, there was no significant variation in fungal richness among the six combinations of the two habitats across the three parks (Figure S1).

**FIGURE 3:**
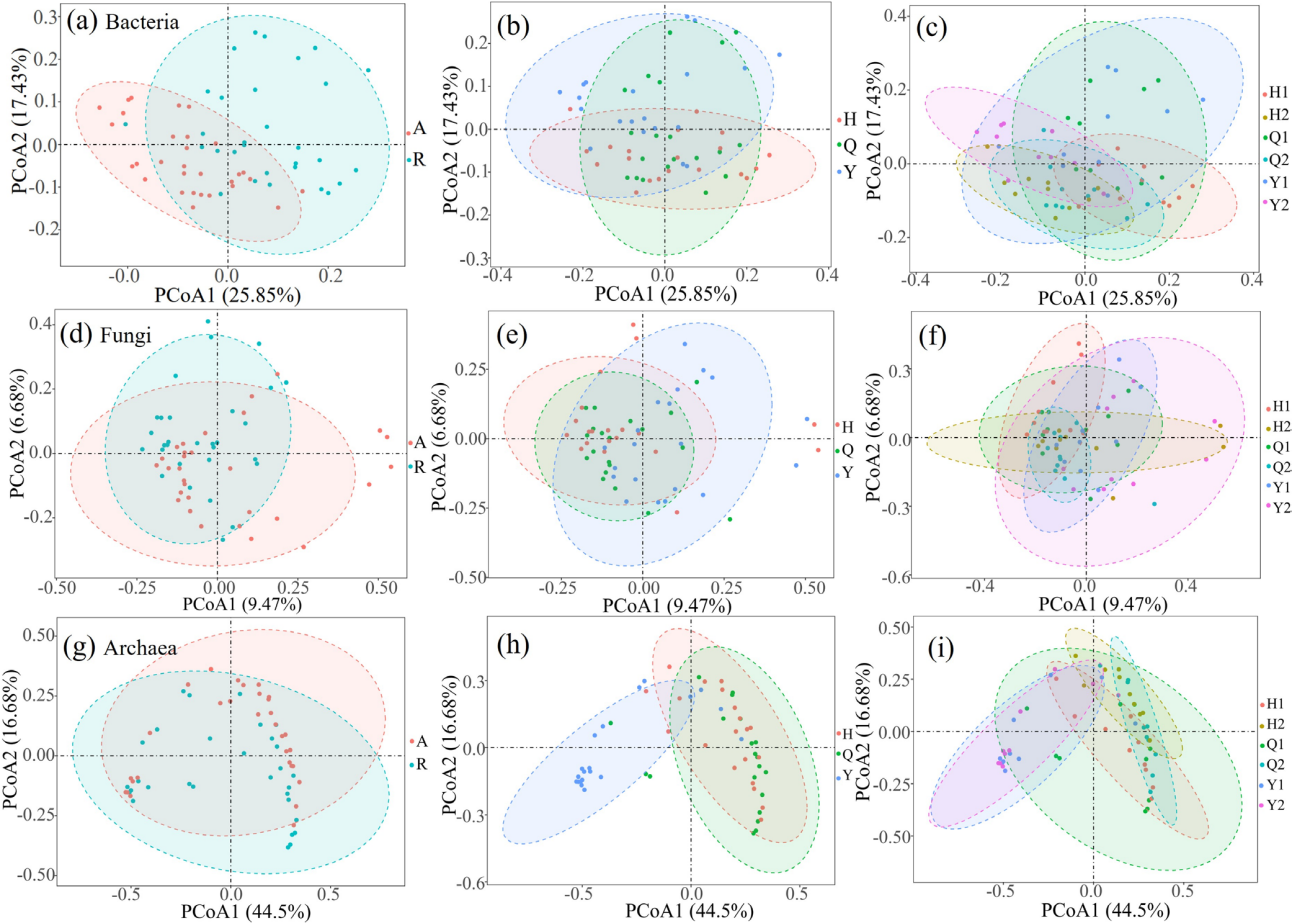
Results of PCoA for comparative assessment of β‐diversity in bacteria, fungi and archaea. (a–c) Bacteria; (d–f) fungi; (g–i) archaea. R represents remnant forest; A represents artificial green space; Q represents Qianlingshan Park; H represents Hauguoyuan Park; Y represents Yuelianghu Park; Q1 represents remnant forests in Qianlingshan Park; Q2 represents artificial green spaces in Qianlingshan Park; H1 represents remnant forests in Hauguoyuan Park; H2 represents artificial green spaces in Hauguoyuan Park; Y1 represents remnant forests in Yuelianghu Park; Y2 represents artificial green spaces in Yuelianghu Park.

Significant differences were noted in the bacterial community compositions between remnant forests and artificial green spaces (Figure 3a). When habitat type is not distinguished, the bacterial and fungal communities of the three parks—Yuelianghu Park, Qianlingshan Park, and Huaguoyuan Park—exhibited considerable similarity (Figure 3b,e). Conversely, the archaeal communities across these parks showed significant differences (Figure 3h). Notably, the bacterial communities of the remnant forest in Huaguoyuan Park and the artificial green space in Yuelianghu Park differed significantly from one another (Figure 3c). Moreover, there is a high similarity in the archaeal communities between artificial green spaces and remnant forests in Yuelianghu Park (Figure 3i).

### 3.2 Taxonomic Composition of Microbial Species

**FIGURE 4:**
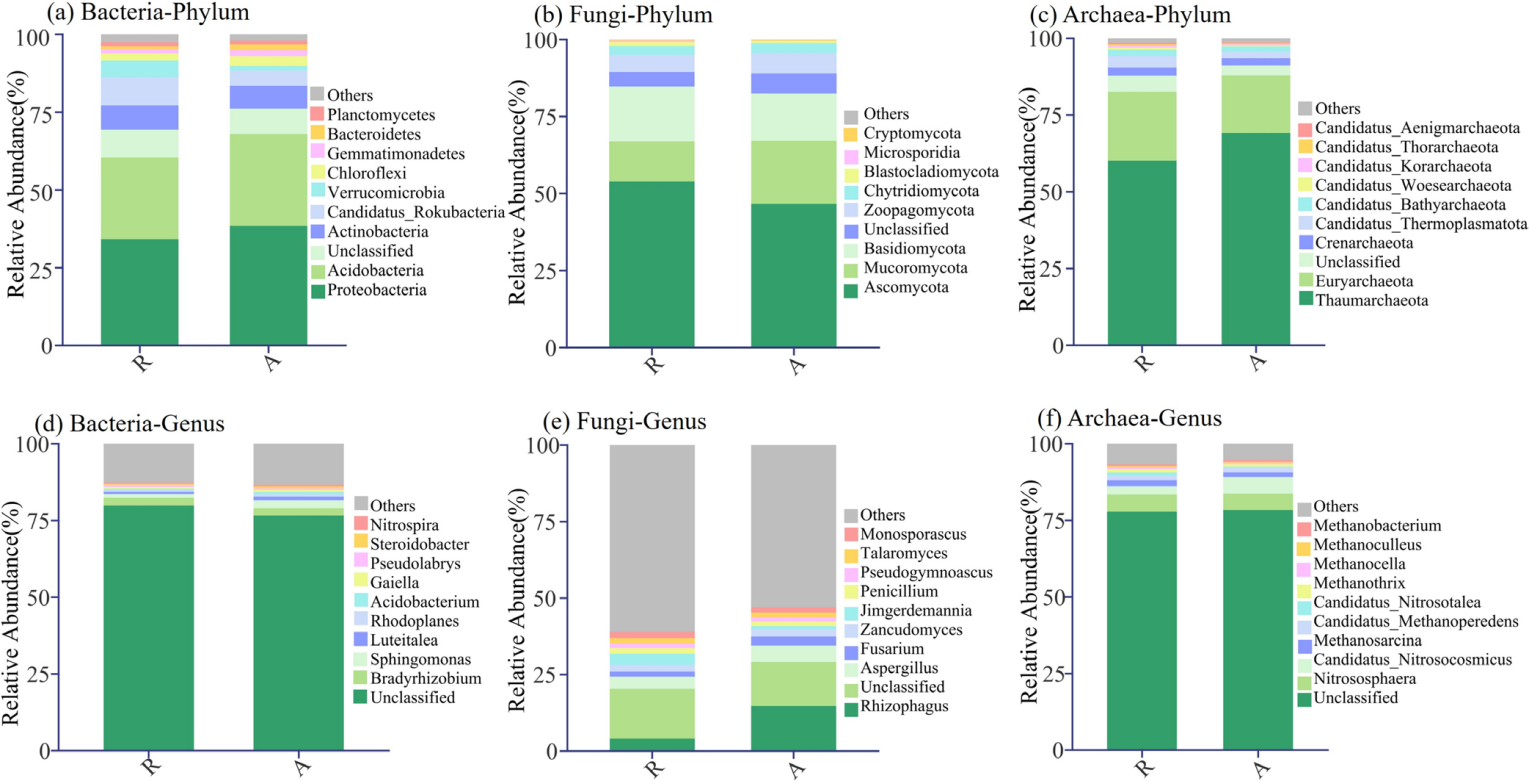
Relative abundance of soil bacteria, fungi and archaea phyla and genera in remnant forest and artificial green space. R represents remnant forest; A represents artificial green space.

The dominant bacterial, fungal, and archaeal phyla and genera were identified in all soil samples, although variations in relative abundance were observed according to habitat types. For bacteria, the phyla Proteobacteria and Acidobacteria showed higher relative abundances in artificial green spaces compared to remnant forests (Figure 4a). Among fungi, the phyla Ascomycota and Basidiomycota exhibited higher relative abundances in remnant forest sites, whereas Mucoromycota was more prevalent in artificial green spaces (Figure 4b). In terms of archaea, the phylum Thaumarchaeota was more abundant in artificial green spaces, whereas Euryarchaeota and unclassified phyla were more prevalent in remnant forests (Figure 4c).

Following the transformation of remnant forest areas in Huaguoyuan Park and Yuelianghu Park, there was a noticeable decrease in the relative abundance of unclassified bacterial genera and an increase in the genus Luteitalea (Figure 4d and Figure S2d). The fungal genus Rhizophagus significantly increased following the habitat transformation (Figure 4e and Figure S2e). Archaeal communities in Qianlingshan Park and Huaguoyuan Park were particularly affected by habitat transitions, with increases in the relative abundance of genera such as Unclassified and Candidatus_Nitrosocosmicu (Figure 4f and Figure S2f).

**FIGURE 5:**
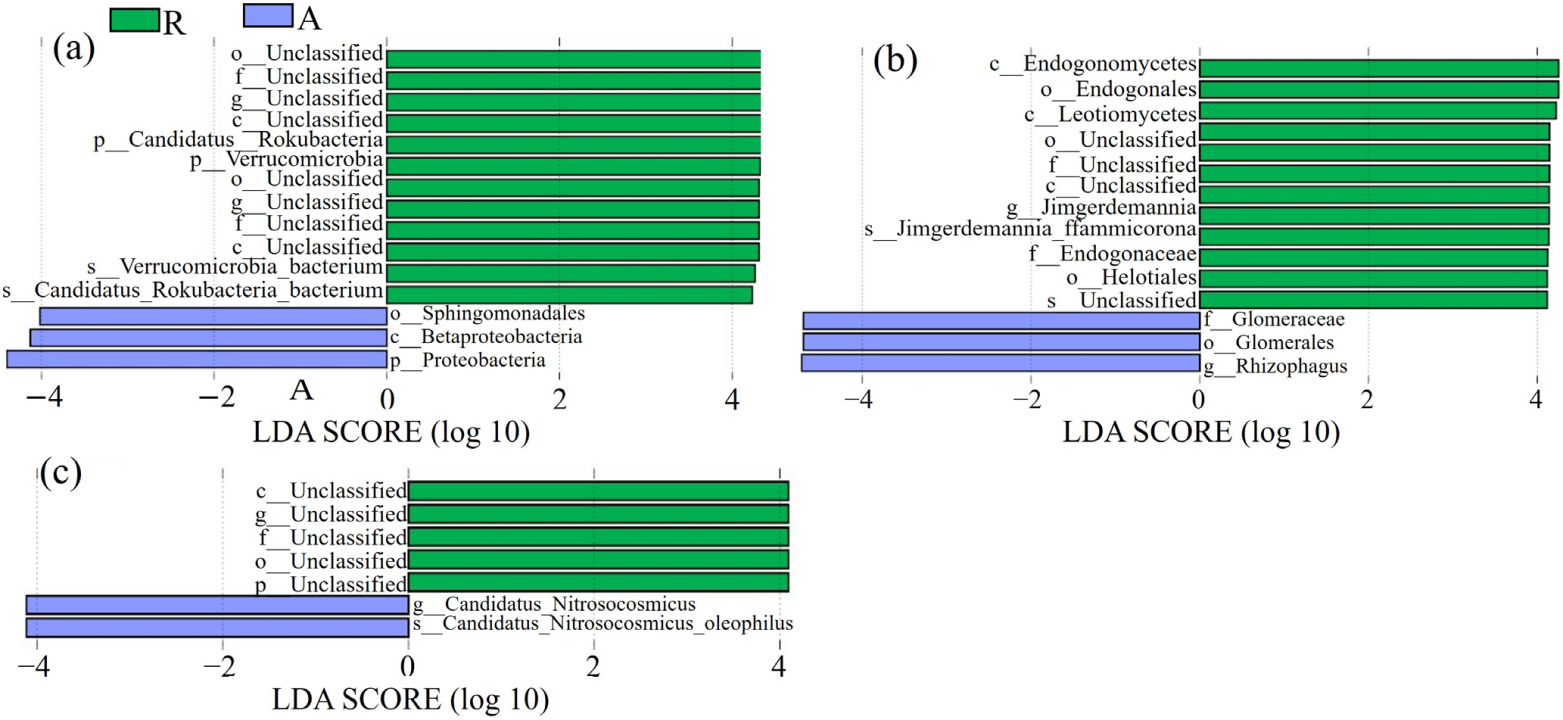
Results of LEfSe for (a) bacterial taxonomic composition, (b) fungal taxonomic composition, and (c) archaeal taxonomic composition. The color of the bar graph distinguishes different sample groups, and the length of the bar graph indicates the magnitude of the LDA score, that is, the relative influence of species shows statistically significant differences between groups (*p* < 0.05).

Based on LEfSe analysis, it was shown that the relative abundance of microbial communities varied according to habitat type. For bacterial, artificial green space was enriched in Proteobacteria (phylum), Betaproteobacteria (class), and Sphingomonadales (order), whereas remaining forests showed significant enrichment at multiple levels in Verrucomicrobia (phylum), Candidatus_Rokubacteria (phylum), and unclassified taxa (Figure 5a). For fungi, Rhizophagus (genus), Glomerulales (order), and Glomerulaceae (family) were enriched in the artificial green space, whereas Jimgerdemannia_flammicorona, Jimgerdemannia (genus), Endogonales (order), and multiple unclassified taxa were enriched in the remnant forest (Figure 5b). For archaea, five biomarkers were significantly enriched in remnant forest, whereas only two biomarkers were enriched in artificial green space. The most discriminative biomarker in artificial green space was the ammonia‐oxidizing archaeon Candidatus_Nitrosocosmicus_oleophilus, followed by its genus Candidatus_Nitrosocosmicus (Figure 5c).

### 3.3 Composition of Functional Genes in Microbes

Compared to the artificial green spaces, remnant forests exhibited a significantly higher abundance of soil microbial genes (Figure S3c). Among the three parks studied, no noticeable differences were observed in the quantity of soil microbial genes (Figure S3b). Huaguoyuan Park's remnant forest demonstrated a markedly greater abundance of soil microbial genes compared to both Qianlingshan Park and the artificial green spaces within Huaguoyuan Park itself. Notably, the artificial green space of Huaguoyuan Park contained significantly fewer soil microbial genes than all other investigated parks and habitat types (Figure S3a).

**FIGURE 6:**
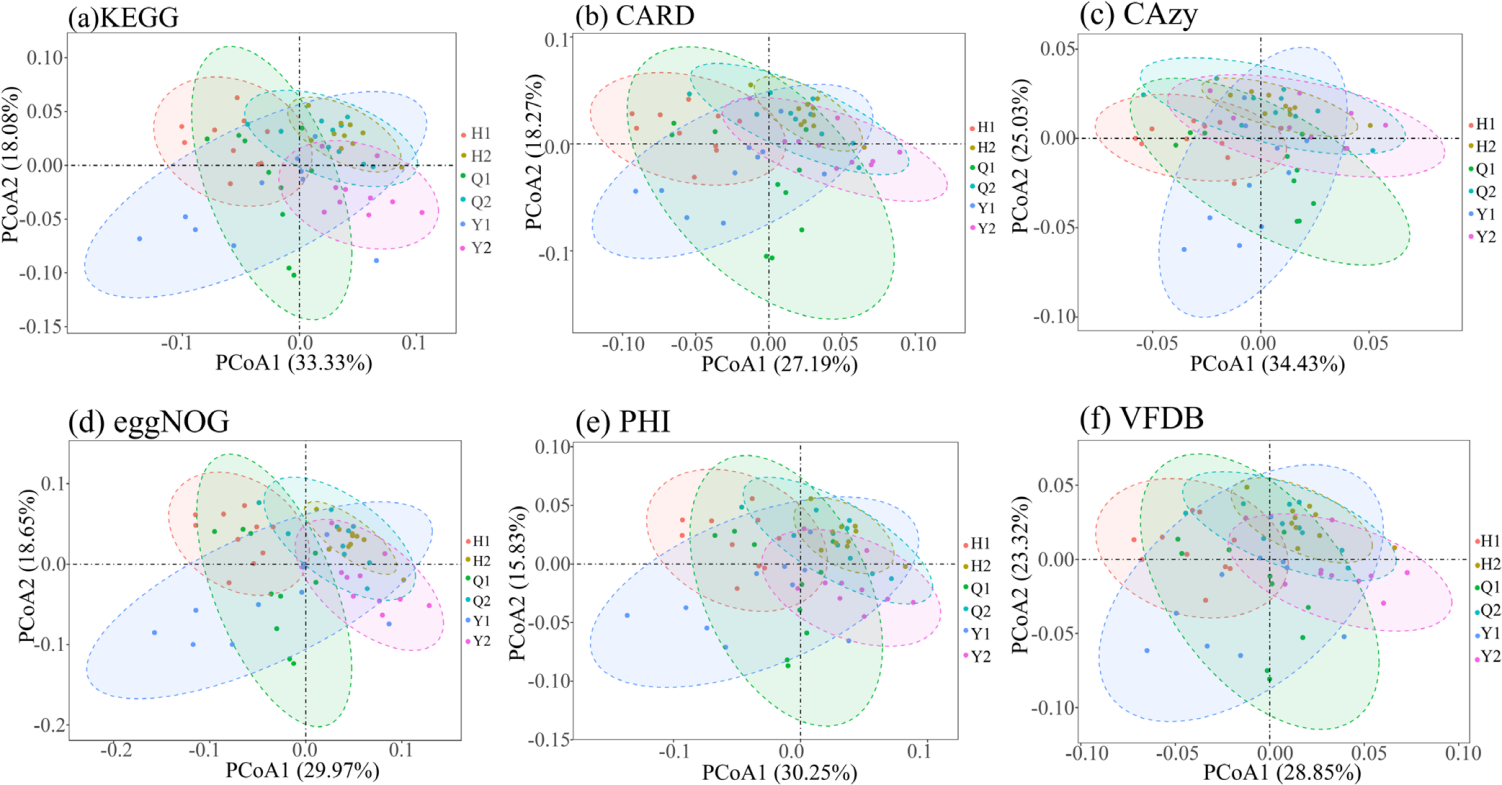
PCoA analyses of functional genes of soil microorganisms. (a–f) KEGG, CARD, CAzy, eggNOG, PHI, VFDB; six combinations of two habitats between the three parks. Q1 represents remnant forests in Qianlingshan Park; Q2 represents artificial green spaces in Qianlingshan Park; H1 represents remnant forests in Hauguoyuan Park; H2 represents artificial green spaces in Hauguoyuan Park; Y1 represents remnant forests in Yuelianghu Park; Y2 represents artificial green spaces in Yuelianghu Park.

Distinct differences were apparent between the remnant forests and artificial green spaces in terms of annotated functional genes associated with various metabolic pathways, antibiotic resistance, carbohydrate‐active enzymes, eggNOG protein functions, genes in PHI predicted to play roles in infection processes, and virulence factors (Figures S4 and S5). Specifically, the remnant forests at Yuelianghu Park and Huaguoyuan Park exhibited the greatest disparity in KEGG annotated functions. High variability in CARD, CAZy, and PHI functional genes was found between the artificial green spaces and the remnant forests within Huaguoyuan Park. Additionally, the artificial green spaces of Yuelianghu Park, along with the remnant forests and artificial green spaces of Huaguoyuan Park, showed considerable variation in VFDB and eggNOG functional genes (Figure 6).

**FIGURE 7:**
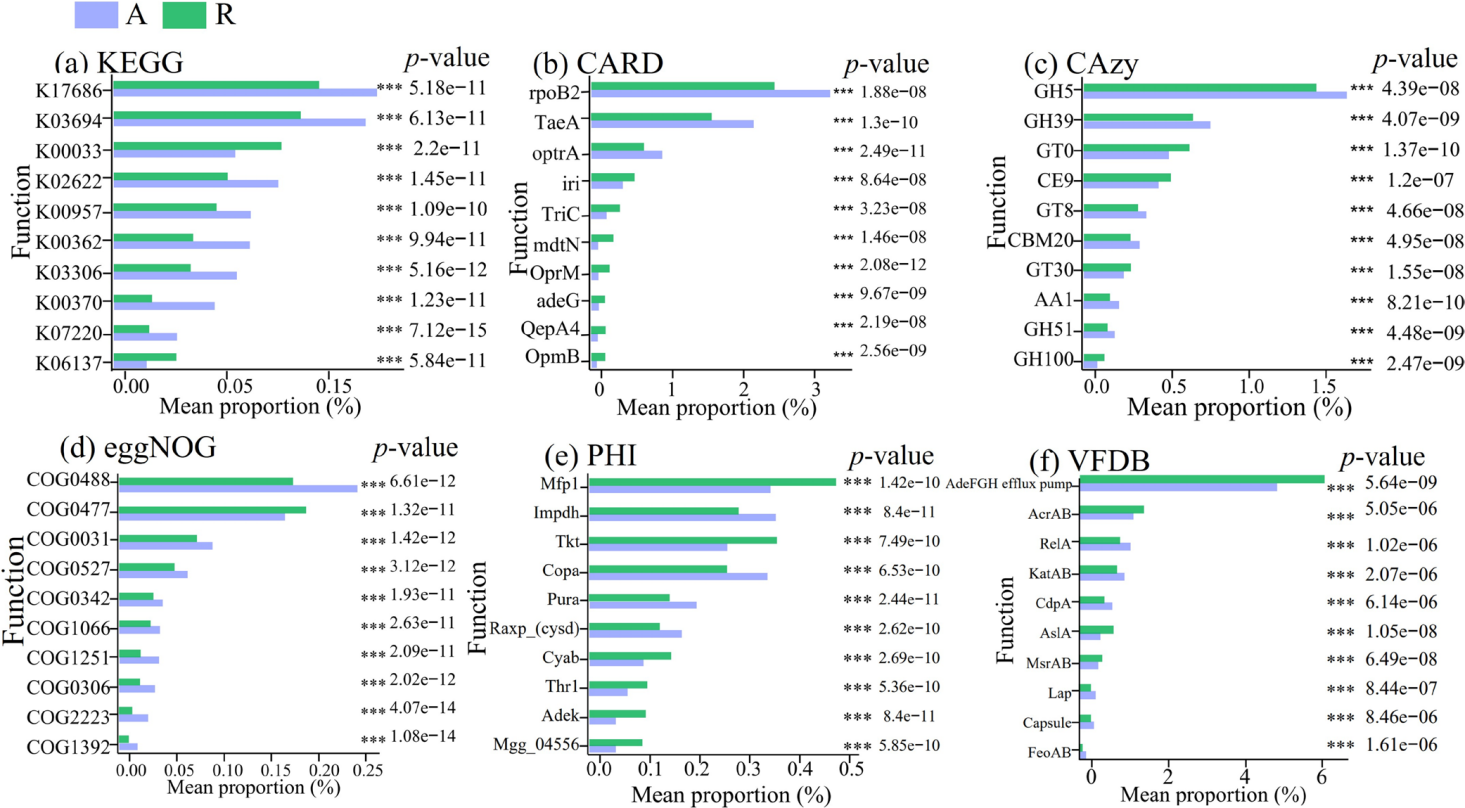
Result of *t*‐test for relative abundance of functional genes among remnant forest and artificial green space in three parks. The leftmost side is a histogram of the mean functional abundance, the horizontal coordinate is the average proportion, the vertical coordinate is the function name; in the middle is the *p* value asterisk mark (*** is less than or equal to 0.0001); the rightmost side is the *p* value; the display is defaulted in accordance with the descending order of the *p* value, then take the first 10, and then in accordance with the abundance of the order from high to low.

In the artificial green space, the relative abundance of eight of the top 10 functional genes relevant to metabolic pathways was significantly greater. Moreover, the top three functional genes related to antibiotic resistance ontology also showed significantly greater relative abundances in this habitat. Additionally, the artificial green space exhibited significantly higher relative abundances of the top two functional genes for carbohydrate‐active enzymes. Conversely, in the remnant forest, the relative abundances of six of the top 10 functional genes associated with proteins predicted to be involved in the process of infection by pathogenic bacteria were significantly higher compared to the artificial green space. Furthermore, the top two functional genes related to virulence factors also exhibited much higher gene abundances in the remnant forest compared to the artificial green space (Figure 7).

### 3.4 Soil Microbial Co‐Occurrence Networks

**FIGURE 8:**
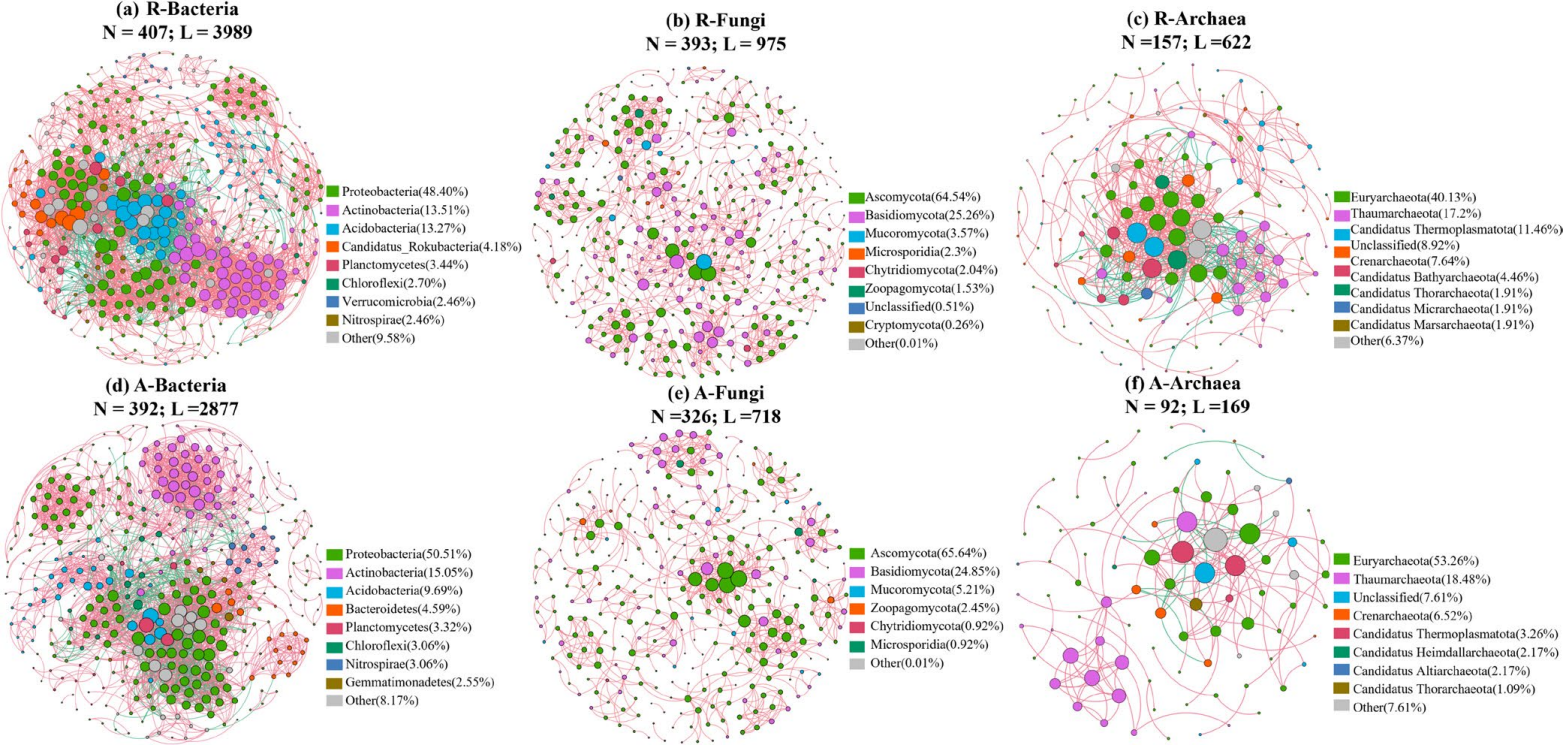
The co‐occurrence network of soil bacteria, fungi and archaea in remnant forest and artificial green space. Each species is represented by a node, the size of which is inversely proportional to the degree of the node; red and green edges represent positive and negative correlations, whereas edges show significant Spearman correlations (*p* < 0.05).

The average path lengths within the co‐occurrence networks of bacteria, fungi, and archaea were found to be longer in the artificial green spaces. Conversely, the remnant forests exhibited higher values in terms of the number of nodes and edges, average degree, connectivity, modularity, and transitivity (Table 1 and Figure 8). Additionally, in artificial green spaces, the betweenness centrality of symbiotic networks was lower for bacterial, fungal, and archaeal populations. Specifically, in the bacterial networks, connectors and module hubs exhibited higher concentrations of Proteobacteria, Actinobacteria, and Acidobacteria. However, in artificial green spaces, the centrality of the Acidobacteria bacterial community was reduced (Figure 8a,d). In the fungal networks, Ascomycota, Basidiomycota, and Mucoromycota displayed higher proportions as connectors and modular hubs, whereas artificial green spaces featured fewer modular hubs (Figure 7b,c). For the archaeal networks, artificial green spaces showed a decrease in connectors and modular hubs, whereas higher proportions of Euryarchaeota and Thaumarchaeota were present in the archaeal networks of remnant forests (Figure 8c,f).

**TABLE 1:** Topological properties of co‐occurrence networks for bacteria, fungi and archaea in remnant forests and artificial green spaces.

Type	Average degree	Modularity	Transitivity	Average path distance	Network diameter	Network density	Clustering coefficient
R‐Bacteria	**19.60**	**0.67**	0.65	3.80	0.00055	**0.048**	0.57
A‐Bacteria	14.68	0.65	0.65	**4.10**	0.00086	0.038	0.58
R‐Fungi	**4.97**	**0.93**	**0.05**	6.82	0.0045	0.013	0.23
A‐Fungi	4.41	0.92	0.04	**6.88**	0.0050	0.014	0.20
R‐Archaea	**7.92**	0.60	**0.55**	2.86	0.0055	**0.051**	**0.57**
A‐Archaea	3.48	0.68	0.41	**3.25**	0.013	0.038	0.44

*Note:* Bold numbers indicate that this value is greater for remnant forests than for artificial green spaces.

## 4 Discussion

Conversion of remnant forests to artificial green spaces resulted in a decrease in the richness of bacteria and archaea, and a non‐significant change in the richness of fungi. Bacteria are strongly associated with environmental variability, whereas the mycelial network that facilitates nutrient uptake by fungi may contribute to their relatively weak sensitivity to environmental change (Guhr et al. 2015; Bahram et al. 2018; Emiru et al. 2018). Vegetation significantly influences soil microbial diversity, and the transition from karst mountain forests to urban parks has led to a homogenization of woody plant composition (Weng et al. 2021; Wang, Gao, et al. 2024; Shafagh et al. 2019). In addition, artificial green spaces are more frequently disturbed and compete more intensely for resources, resulting in fewer available niches, lower microbial abundance, supporting fewer adaptive microbial species, and more homogeneous microbial communities (Sébastien et al. 2017; Brinkmann et al. 2019; DelgadoBaquerizo et al. 2021). In a study of three parks in Guiyang, the soil bacterial composition of the artificial green spaces and remnant forests in all three parks was dominated by Proteobacteria and Acidobacteria phyla, and the soil fungi were dominated by Ascomycota phylum, similar to that found in many other cities (Hu et al. 2018; Gill et al. 2020; Baruch et al. 2020; Amélie et al. 2023). This evidence suggests that there are no significant differences in the core soil microbial diversity between artificial green spaces and remnant forests. Remnant forests without soil tillage, the presence of permanent plant cover, and a longer historical continuity (Buil et al. 2021). These factors contribute to significant inputs of root secretions, which increase soil moisture and nutrient content in less disturbed environments (Bonfante and Venice 2020; Romdhane et al. 2022). Enrichment of Verrucomicrobia and unclassified taxa in remnant forests may indicate adaptation to niche specific conditions. In contrast, artificial green spaces are subject to a variety of management practices that alter soil chemistry and, consequently, microbial abundance (Baruch et al. 2020).

The conversion of remnant forests to artificial green spaces may result in alterations in plant–soil microbial interactions, leading to a decrease in microbial species abundance alongside an increase in relevant functional genes (Peng et al. 2024). This shift may explain the rise in dominant genes associated with metabolic pathways. Artificial greenery usually requires regular interventions such as fertilization and pruning, and due to the continuous supply of nutrients, it usually accommodates soil microbial communities rich in metabolic pathway genes (Luo et al. 2023); in contrast, the vegetation and organic matter of remnant forests are more diverse and support a wider range of microbial functions, so the metabolic genes of artificial greenery space are increased (Huang et al. 2019). In addition, residual effects of past land use on soil microbial communities vary widely in duration (Jangid et al. 2011; Romdhane et al. 2022). The microbial functional genes of Yuelianghu Park and Huaguoyuan Park had a high degree of similarity, whereas the functional genes of the two parks differed significantly from those of Qianlingshan Park. The disparity could potentially be attributed to the time of park renovations: Yuelianghu Park and Huaguoyuan Park completed their renovations in 2020 and 2010, respectively, whereas Qianlingshan Park renovation was completed in 1957. Prolonged forest cover leads to changes in soil properties (e.g., C and N content or pH), which are important drivers of functional genes in soil microbial communities (Crème et al. 2018; Yang et al. 2024). Similar to findings from a study on urban soils in Tianjin, China, urban green space exhibited a higher abundance of resistance genes (Meng et al. 2024). The structure of bacterial communities for the spread of antibiotic resistance is largely influenced by plant and soil properties (Moreau et al. 2019; Zheng et al. 2022). Artificial green soils are often modified by the removal or addition of fertilizers and organic materials, and such management practices alter the microbial composition of the soil and promote the development and spread of antibiotic resistance genes (Liu et al. 2023). Frequent irrigation and maintenance activities further enhance the mobility and spread of these genes within the soil matrix (Charlotte et al. 2023). Moreover, the nutrient‐rich conditions prevalent in artificial environments create conducive ecosystems for microbes harboring resistance genes to thrive (Dai et al. 2023). In contrast, high microbial biodiversity in remnant forests regulates the spread of certain microbial populations containing antibiotic resistance genes through natural mechanisms such as competition and predation (Wang, Xu, et al. 2024).

The ecological dynamics of ecosystems subject to frequent disturbance differ fundamentally from those of stable ecosystems (Karimi et al. 2019). Enhanced soil management practices in artificial green spaces promote the diversification of microbial niches (Lupatini et al. 2014; Schlatter et al. 2015). Taking into account the functional redundancy hypothesis, the abundance of microbial species in artificial green space decreases, and a few metabolically flexible species become dominant with more functional gene abundance. Members of the fungus phylum Ascomycota can establish symbiotic relationships with plants, which may reduce interactions within and between microbial communities (Morris et al. 2013; Egidi et al. 2019). Under the same management conditions, the ability of remnant forest to resist changes and recover to its original state was stronger than that of artificial green space, so remnant forest had a more complex microbial symbiotic network than artificial green space. Furthermore, the conversion of remnant forests into artificial green spaces decreases the abundance of microbial species, leading to the fragmentation of central clusters in the microbial network and thus altering its structure.

Despite increasing our understanding of how the species composition and functional gene diversity of soil microorganisms are impacted by the conversion of remnant forests into urban parks, our study has limitations. Our analysis focuses only on the microbial community structure itself. Failure to incorporate other key variables, especially key soil physicochemical properties (e.g., pH, organic matter content), as well as important human management practices (e.g., frequency and intensity of irrigation, fertilization) and plant species selection, all of which may significantly affect microbial functional characteristics, is a limitation. Future research is urgently needed in conjunction with soil physicochemical analysis to assess the relative contributions of these variables (including management practices, plant selection and soil physicochemical properties) to microbial communities. Another limitation is the regional focus on karst mountain cities, leaving unanswered whether our findings are applicable to urban parks in other settings such as cities on plains, or to the conversion of different types of land. Thus, broader, more comprehensive studies are needed to generalize our findings across different urban and ecological environments.

## 5 Conclusions

This study explored the ecological impact of transforming a karst mountain remnant forest into an urban park, focusing specifically on alterations in soil microbial species composition and functional gene diversity. We discovered that the transformation led to a notable increase in the relative abundance of dominant functional genes associated with microbial metabolic pathways, carbon‐hydroxylate‐activated enzymes, and antibiotic–antibody ontologies within the urban park. However, this was contrasted by a significant reduction in microbial richness compared to the natural forest setting. Additionally, the remnant forest showcased more complex co‐occurrence networks among bacteria, fungi, and archaea, suggesting a more interconnected and possibly resilient ecosystem. The findings indicate that while artificial green spaces can support microbial communities with enhanced metabolic versatility, the reduction in microbial richness and abundance may undermine broader ecological functions. This underscores the essential role of soil microbial health in ecosystem sustainability as we reshape natural landscapes into urban environments. The integration process prioritizes the preservation of native vegetation, emphasizing that the construction of recreational facilities and the use of organic soil amendments or chemical inputs should be minimized in areas with remnant forest soils.

## Author Contributions

**Chunhua Cen:** data curation (equal), investigation (equal), methodology (equal), software (equal), validation (equal), visualization (equal), writing – original draft (equal), writing – review and editing (equal). **Weize Wang:** investigation (equal), methodology (equal), writing – review and editing (equal). **Mengping Jian:** investigation (equal), methodology (equal), writing – review and editing (equal). **Zijin Wang:** investigation (equal), methodology (equal), writing – review and editing (equal). **Jingyi Yang:** conceptualization (equal), data curation (equal), formal analysis (equal), funding acquisition (equal), project administration (equal), resources (equal), supervision (equal), writing – review and editing (equal).

## Ethics Statement

The authors have nothing to report.

## Conflicts of Interest

The authors declare no conflicts of interest.

## Supporting information


**Data S1:** ece372021‐sup‐0001‐Supinfo.docx.

## Data Availability

The data for this study are available via the Mendeley Data Repository. https://doi.org/10.17632/3hrcfpjbsr.1.
