# Supplementary material for: Impact of Habitat Transformation on Soil Microbial Diversity and Functionality in Karst Mountainous Parks: A Comparative Study of Remnant Forests and Artificial Green Spaces
Source: Ecol Evol. 2025 Aug 14;15(8):e72021. doi: 10.1002/ece3.72021 (PMC12352970; doi:10.1002/ece3.72021)
Supplement: Supplementary file 1 — Data S1: ece372021‐sup‐0001‐Supinfo.docx. [file ECE3-15-e72021-s001.docx]

# Electronic supplementary material


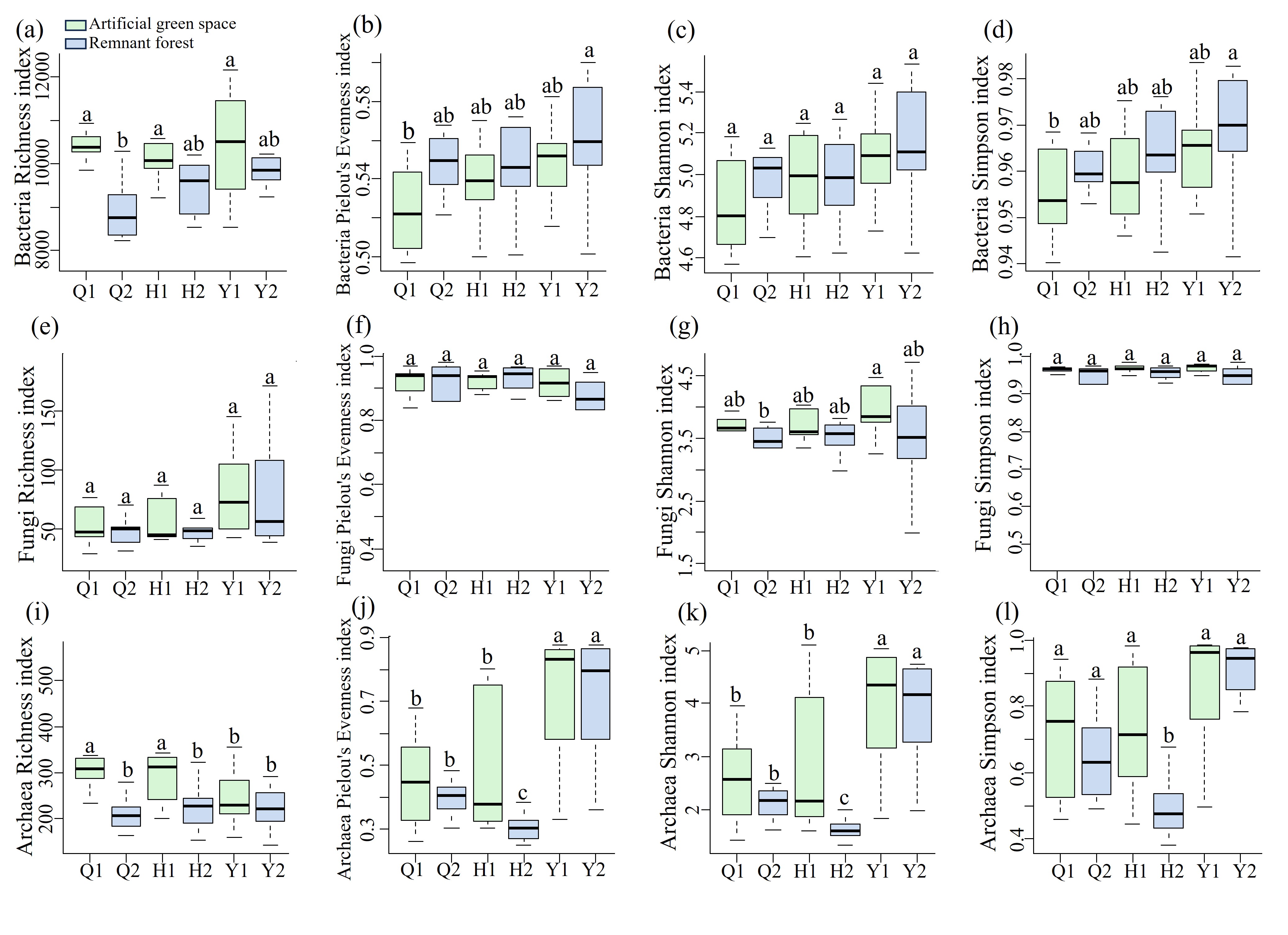


Fig. S1 Analysis of microbial species α-diversity among six assemblages of three parks and two habitat types. (a-d) Bacteria; (e-h) Fungi; (i-l) Archaea.


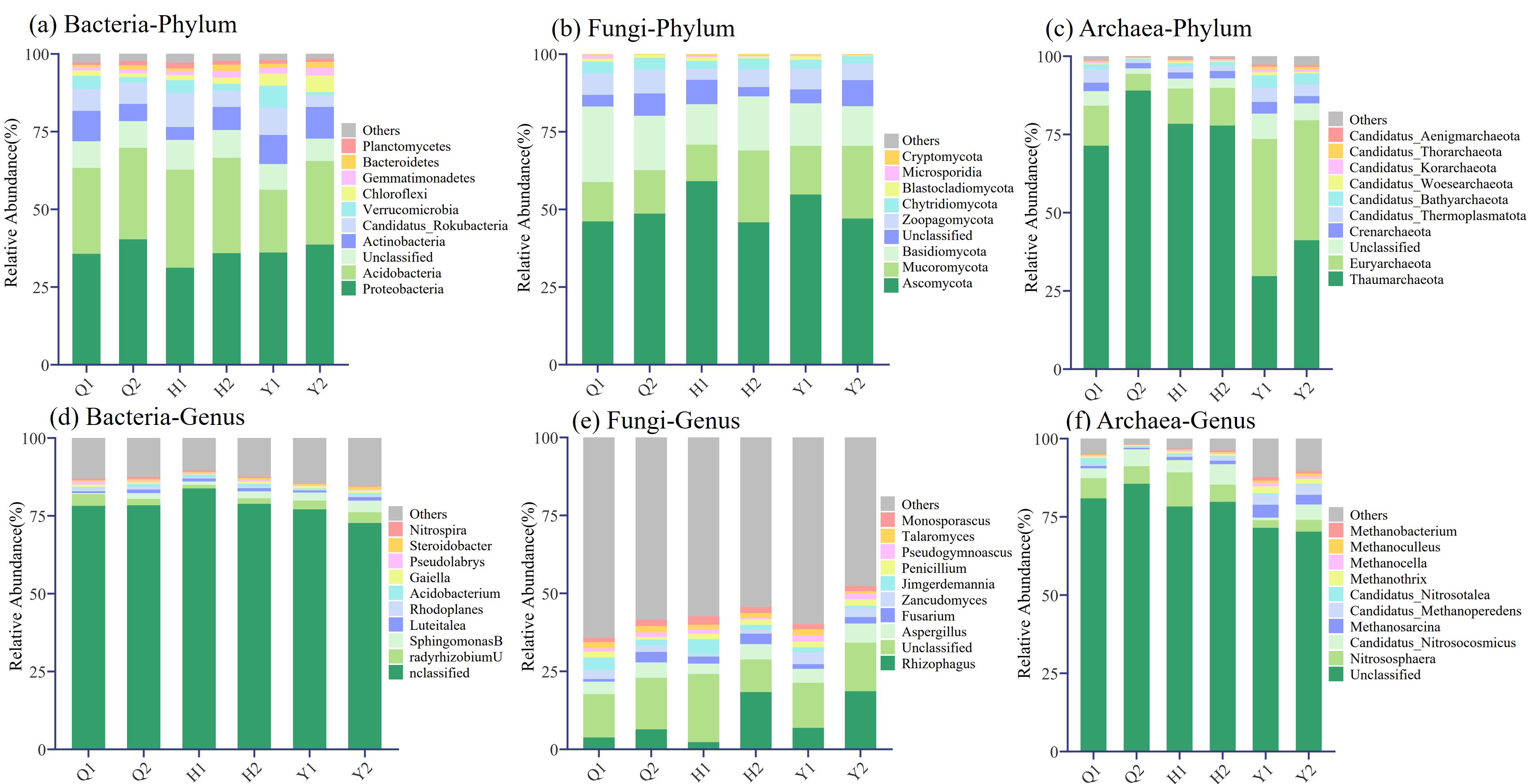


Fig. S2 Relative abundance of phyla and genera of soil Bacteria, Archaea, Fungi


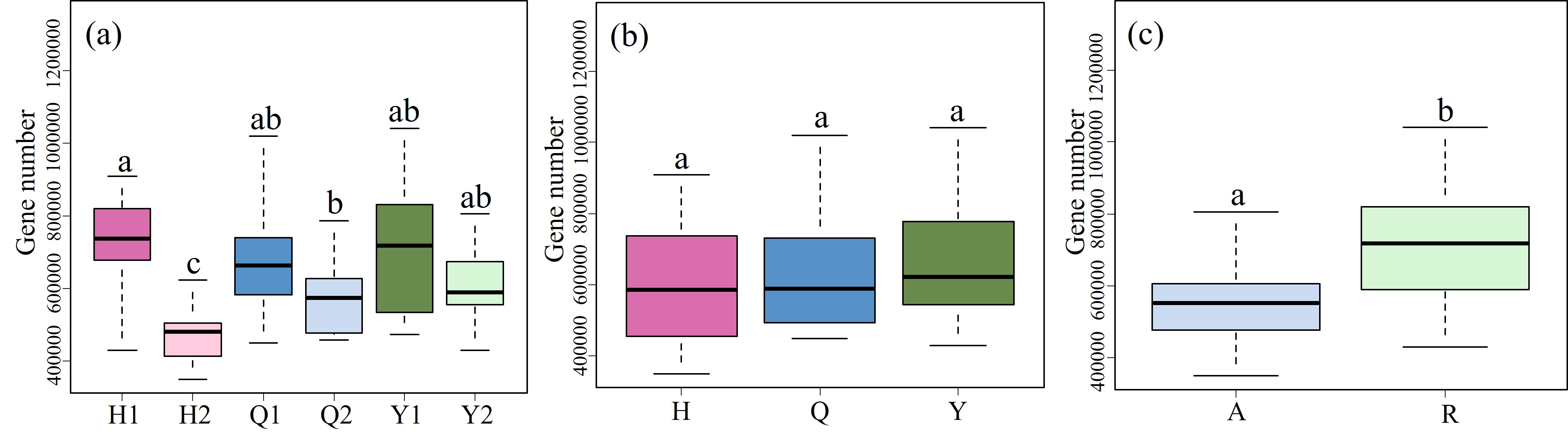


Fig. S3 Differences in the number of soil microbial genes


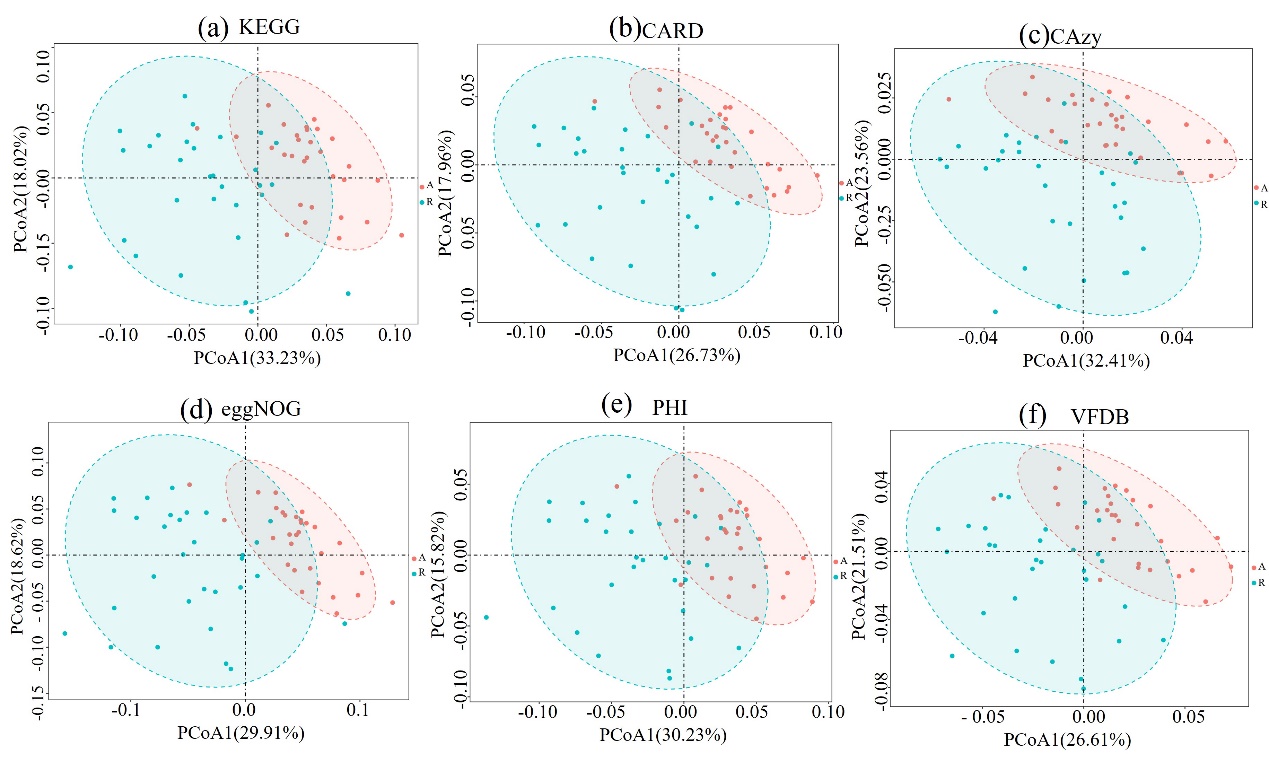


Fig. S4 Microbial functional gene PCoA in two habitats, remnant forest and artificial green space. A: artificial green space; R: remnant forest.


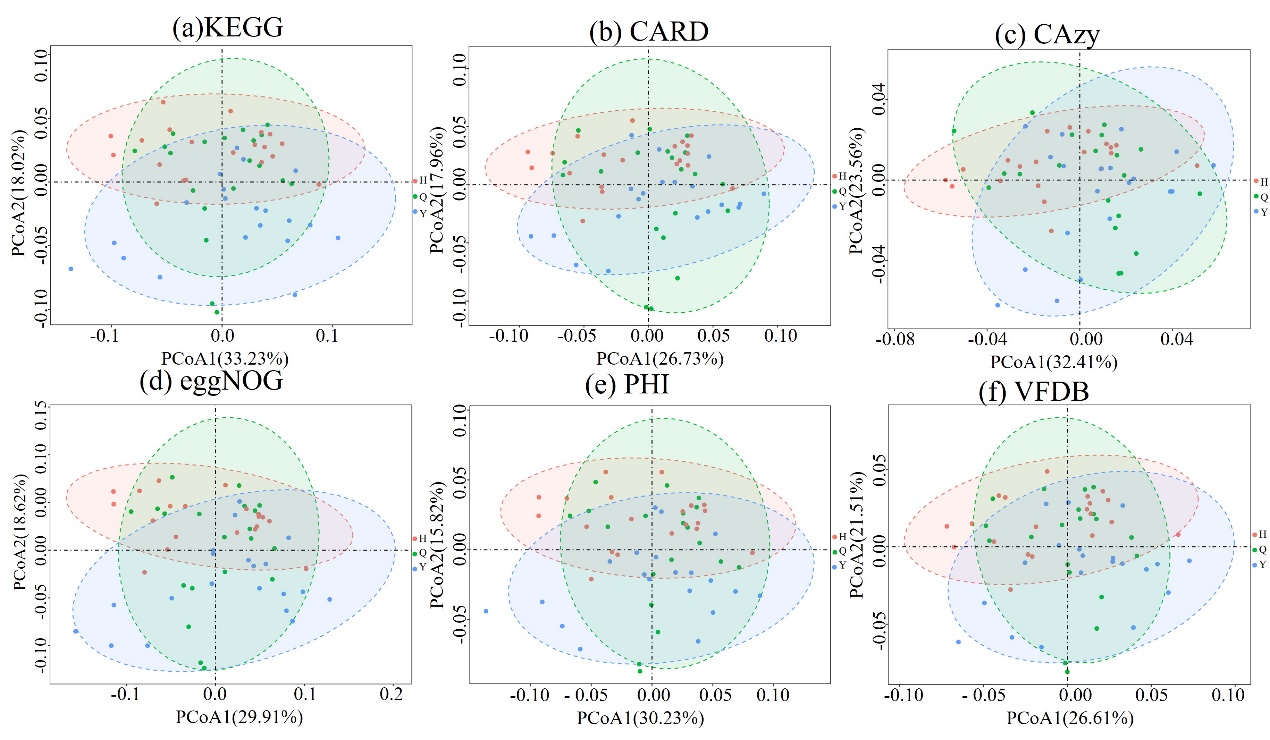


Fig. S5 Results of PCoA analyses of microbial functional genes in three parks, H: Huaguoyuan park; Q: Qianlingshan park; Y: Yuelianghu park.

Table S1 Basic information of three parks.

| Park | Established  time | Remaining forest area (km^2^) | Artificial green space area (km^2^) | Major plant species |
| --- | --- | --- | --- | --- |
| Qianlingshan Park | 1957 | 4.0546 | 0.2235 | *Itea yunnanensis; Lindera communis; Camellia oleifera; Myrsine Africana; Osmanthus fragrans; Cerasus yedoensis* |
| Huaguoyuan Park | 2010 | 5.473 | 0.1826 | *Itea yunnanensis; Lindera communis; Alangium chinense; Rosa cymose; Osmanthus fragrans; Cerasus yedoensis* |
| Yuelianghu Park | 2020 | 0.9499 | 2.9021 | *Itea yunnanensis; Populus adenopoda; Corylus heterophylla; Myrsine Africana; Osmanthus fragrans* |
